# Ultrasound assessment of tensile stress in carotid arteries of healthy human subjects with varying age

**DOI:** 10.1186/s12880-019-0394-5

**Published:** 2019-11-29

**Authors:** Xianghong Luo, Lianfang Du, Zhaojun Li

**Affiliations:** 1 Department of Echocardiography, Shanghai General Hospital, Shanghai Jiao Tong University School of Medicine, Shanghai, 200080 China; 2 Department of Ultrasound, Shanghai General Hospital, Shanghai Jiao Tong University School of Medicine, 100 Haining Road, Hongkou District, Shanghai, 200080 China

**Keywords:** Shear wave dispersion, Shear wave elastography, Tensile stress, Carotid artery, Viscoelasticity, Ultrasonography

## Abstract

**Background:**

Arterial remodeling is thought to reflect the adaptation of the vessel wall to mechanical and hemodynamic stimuli and contributes to the progression of cardiovascular and cerebrovascular diseases. Tensile stress (TS) is one of the mechanical properties of the artery wall. The purpose of this study was to investigate the tensile stress change (TS) of carotid artery with varying viscoelasticity in healthy subjects within two groups of different ages.

**Methods:**

Forty-five subjects were recruited and randomly assigned into the group at the age above 50 years and below 50 years. The carotid arteries were examined by ultrasonography, using the techniques of shear wave elastography (SWE), shear wave dispersion (SWD) and radiofrequency (RF) -based ultrasound. The following values, including elastic modulus (SWER) and viscous index (SWDR), as well as the peak and mean TS of the left and right carotid arteries (L-PTS, R-PTS, L-MTS and R-MTS) were measured. The correlations between SWER, SWDR and tensile stress were evaluated.

**Results:**

The SWE_R_ and SWD_R_ of carotid arteries are lower in the subjects ≥50 years old than the subjects younger than 50 years (SWE_R_, 10.29 ± 9.57 kPa VS 17.24 ± 14.07 kPa; SWD_R,_ 11.99 ± 3.51 (m/s)/kHz VS 13.97 ± 3.71 (m/s)/kHz, *P* < 0.05). The R-PTS was lower in the group with younger age (*P* < 0.05). Pearson correlation analysis showed that SWE_R_ of carotid artery was positively correlated with the parameters of tensile stress, R-PTS, R-MTS, L-PTS and L-MTS(*r* = 0.218, *r* = 0.359, *r* = 0.209 and *r* = 0.369, respectively, *P* < 0.05). However, SWD_R_ of carotid arteries was not significantly associated with TS.

**Conclusion:**

Ultrasonic shear wave imaging could be used to quantitatively assess carotid viscoelasticity. The carotid TS was related to its elasticity while little related to its viscosity, suggesting that mechanical properties of the arterial wall might be better revealed.

**Trial registration:**

Date of our trial registration: 2018-06-11. Registered with the official website of China Clinical Trial Registration Center (ChiCTR1800016590)

## Background

Cardiovascular disease is still a major cause of morbidity and mortality in the world. It is essential to recognize early changes in vascular function and morphology to help identify individuals at risk for cardiovascular disease. Arterial remodeling or dilatation of carotid arteries has been shown to be related to increased mechanical stress from high pulsatile loads, leading to breakdown of elastic fibers within the arterial wall [1, 2]. Arterial remodeling is a potentially important pathophysiologic change in the development of atherosclerosis, which acts to counteract the development of lumen compromise of large artery by mutual adaptation of diameter to wall thickening [3]. Carotid geometry and wall tensile stress can be evaluated at the carotid artery, because its simple geometry allows the application of Laplace’s law for wall stress estimation. Hypertension increases the tensile stress applied on the carotid artery, thus carotid intima-media thickness (IMT) and stiffness, and favors atherosclerotic plaque progression [4].

The arterial wall has been known as one of the most intricate structures and presents a certain biomechanical property of viscoelasticity [5]. This behavior presents a non-linear mechanical relationship and attributes in part to fluid transport within the solid matrix [6, 7]. However, most of the arterial TS had been measured through biomechanical experiments in vitro, and little is known about the nature of the adjustments to the arterial viscoelasticity, which corresponds to the altered wall TS.

In recent years, shear wave-based elastography techniques, such as shear wave elastography (SWE) and shear wave dispersion (SWD) have received wide attention for noninvasive assessment of elasticity and viscidity properties [8, 9]. This study was to assess the carotid TS and viscoelasticity by ultrasonic technologies and explored the relationship between them.

## Methods

### Study design and setting participants

The study was registered as a clinical trial (ChiCTR1800016590, 2018-06-11) approved by our Institutional Ethics Review Board (2017KY009). Written informed consent was obtained from all participants in this study.

Forty-five healthy subjects were enrolled from August 1, 2017 to May 1, 2018. They were selected based on the electronic medical records. Participants that have active bleeding, history of cardiovascular or cerebrovascular events, vascular diseases of the extremities, immune diseases, severe liver, lung, kidney diseases, or malignant tumor were excluded. They were divided into 2 groups according to their ages: group A (≥50 years) and group B (< 50 years). Blood pressure was measured in the supine position after 10 min of rest and defined as the average of 3 consecutive systolic blood pressure (SBP) and diastolic blood pressure (DBP). Blood samples were obtained for the measurements of glucose, high density lipoprotein (HDL), triglycerides and low-density lipoprotein-cholesterol (LDL) after 12 h of fasting.

### Carotid artery B-mode ultrasound

**Fig. 1:**
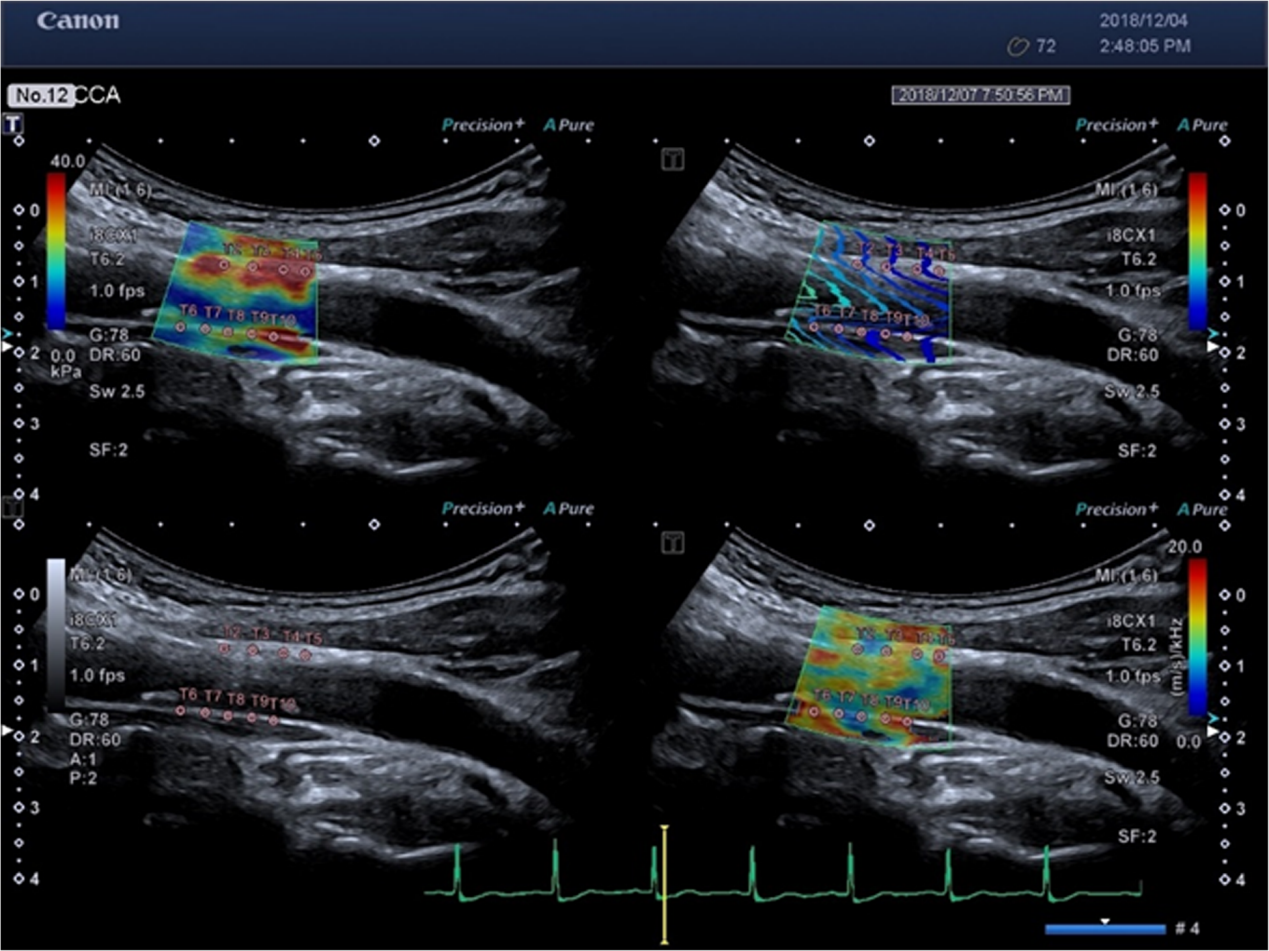
The arterial shear wave profiles were displayed by. QuadView, including 4 maps for: **a** elastic map, **b** propagation map, **c** two-dimensional reference map and **d** shear wave dispersion map.

After high-resolution common carotid artery (CCA) images were obtained, the viscoelasticity of the CCA was measured using an Aplio 900 ultrasound system (Canon Medical Systems Corporation, Otawara, Japan) equipped with PVI-475BX curved abdominal transducer (frequency range:1–8-MHz and mid frequency: 5.0 MHz) [10]. The ultrasound image was frozen at electrocardiographic end diastole (R-wave) by electrocardiogram (ECG) triggering. Five 2 mm circular regions of interest (ROI) were selectively placed on anterior and posterior walls of bilateral carotid arteries (1 cm proximal to the carotid bifurcation). The motion of vascular wall for 10–20 cardiac cycles was recorded and measurements were taken during the systolic phase. Shear wave elastic modulus (SWE_R_) and shear wave dispersion (SWD_R_) were measured after the R wave of the ECG. The arterial shear wave profiles, including the elastic map (Fig. 1a), propagation map (Fig. 1b), two-dimensional reference map (Fig. 1c) and shear wave dispersion map (Fig. 1d) were obtained with single-shot acquisition and displayed on QuadView.

The carotid structure and tensile stress were measured using Mylab Twice ultrasound system (Esaote, Firenze, Italy) equipped with a LA523 linear transducer (4–13 MHz) and the software, which enables the analysis of radiofrequency-based ultrasound images. The common carotid intima-media thickness (CIMT) and inner diameter (CCID) were measured by radiofrequency-based ultrasound tracking of the carotid wall displacement for at least six cardiac cycles and the mean values were calculated automatically by the software. The carotid peak tensile stress (PTS) and mean tensile stress (MTS) were calculated according to Laplace’s law using the equations: (PTS=SBP×(CCID_R_/2)/CIMT (mmHg), MTS = MBP×(CCID_T_ /2)/CIMT (mmHg) [11]. SBP was systolic blood pressure, while MBP was the mean blood pressure and calculated by (SBP + 2 × DBP)/3. CCID_R_ was the inner diameter of common carotid artery at the end of systolic phase, while CCID_T_ was the inner diameter of common carotid artery at the end of diastolic phase. 1dyne/cm^2^ = 7.5 × 10^− 4^ mmHg.

### Statistical analyses

All statistical analyses were performed using SPSS 13.0 statistical analysis software. The continuous variables were expressed as mean ± SD and compared using Student’s *t*-tests, while categorical variables were expressed as percentages and compared using chi-squared test. Agreement of measurement was evaluated by Bland-Altman plots. Pearson correlation analysis was used to evaluate the correlation between carotid viscoelasticity and TS. *P* < 0.05 was considered statistically significant.

## Results

### Clinical characteristics

The baseline characteristics of two age groups were summarized in Table 1. The mean height of the subjects in group A is less than that of group A. The mean SBP and DBP were higher in group A (all *P* < 0.05) than group B. No significant difference of gender ratio, weight, body mass index, fasting glucose, TG, TC, LDL-C was found between the two groups (all *P* > 0.05).

**Table 1:** Characteristics of two age groups

Variable	Group A (*n* = 23)	Group B (*n* = 22)	*t* /χ2	*P*
Gender (F/M)	11/12	11/11	0.023	0.879
Height, cm	162.9 ± 7.1	167.5 ± 7.9	−2.301	0.025
Weight, kg	68.3 ± 17.3	66.7 ± 15.6	0.348	0.729
Body mass index, kg/m^2^	25.8 ± 7.4	23.5 ± 4.1	1.444	0.154
SBP, mmHg	139.6 ± 11.1	125.7 ± 9.1	5.102	<0.001
DBP, mmHg	87.9 ± 7.2	79.7 ± 6.1	4.607	<0.001
Glucose, m mol/L	6.1 ± 1.61	5.2 ± 1.21	1.001	0.327
Total cholesterol, mmol /L	4.6 ± 1.11	4.4 ± 0.81	0.763	0.456
Triglycerides, mmol/L	1.7 ± 1.31	1.2 ± 0.81	1.12	0.275
Low-density lipoprotein, mmol/L	2.9 ± 1.01	2.7 ± 0.81	0.77	0.433
Diabetes mellitus (*n*)	3	1	0.156	0.693
Hypertension (*n*)	5	3	0.044	0.833

1 mmHg =0.133 kPa

### Geometry, viscoelasticic feature and tensile stress of carotid arteries in two groups

The SWE_R_ and SWD_R_, which are the two parameters that represent the carotid viscoelasticity, were significantly lower in the group A than those in the group B (*P* = 0.040 and 0.043, respectively). The bilateral CIMT, CCID_T_ and CCID_R_, which are used to characterize the geometry of the carotid arteries, were all higher in the group of subjects older than 50 years (all *P* < 0.05). The mean PTS of the right carotid arteries was lower in the group A (*P* < 0.05), while no significant differences of the PTS of left carotid arteries and bilateral MTS were found between the two groups (all *P*>0.05) (Table 2).

**Table 2:** Comparison of carotid viscoelasticity, structure and tensile stress between groups

Variable	Group A (*n* = 23)	Group B (*n* = 22)	*t*	*P*
Viscoelasticity				
SWE_R_ kPa	10.29 ± 9.57	17.24 ± 14.07	−2.236	0.040
SWD_R_ (m/s)/kHz	11.99 ± 3.51	13.97 ± 3.71	−2.129	0.043
Structure				
R-CIMT (μm)	677.6 ± 138.4	449.1 ± 131.9	6.321	<0.001
L-CIMT (μm)	674.7 ± 119.8	503.9 ± 193.2	3.976	<0.001
R-CCID_T_ (mm)	8.23 ± 0.89	6.43 ± 0.40	5.946	<0.001
L-CCID_T_ (mm)	7.93 ± 0.99	6.50 ± 0.41	7.575	<0.001
R-CCID_R_ (mm)	8.57 ± 1.03	7.00 ± 0.55	7.095	<0.001
L-CCID_R_ (mm)	8.20 ± 1.09	7.04 ± 0.56	5.000	<0.001
R-CCAD (μm)	339.9 ± 142.7	571.1 ± 148.2	−5.946	<0.001
L-CCAD (μm)	273.6 ± 98.6	535.4 ± 153.9	−7.575	<0.001
Tensile stress				
R-PTS (mmHg)	920.4 ± 160.3	1073.6 ± 285.8	−2.474	0.017
R-MTS (mmHg)	783.8 ± 189.9	884.4 ± 224.0	−1.812	0.076
L-PTS (mmHg)	648.1 ± 117.4	723.8 ± 195.5	−1.758	0.084
L-MTS (mmHg)	600.9 ± 108.2	644.6 ± 168.2	−1.156	0.253

*SWE*_*R*_ Shear wave elastic modulus in electrocardiographic R wave, *SWD*_*R*_ Shear wave dispersion in electrocardiographic R wave, *L-CIMT* Left common carotid intima-media thickness, *R-CIMT* Right common carotid intima-media thickness; *L-CCID*_*T*_ *and R-CCID*_*T*_ Left and right common carotid inners at end of diastole, respectively, *L-CCID*_*R*_ *and R-CCID*_*R*_ Left and right common carotid inner diameters at end of systole, respectively, *L-CCAD and R-CCAD* Difference of CCID between diastole and systole on left and right, *R-PTS and R-MTS* Right carotid peak tensile stress and mean tensile stress, *L-PTS and L-MTS* Left carotid peak tensile stress and mean tensile stress

1 dyne/cm^2^ = 7.5 × 10^−4^ mmHg

### Correlation between the carotid viscoelasticity and tensile stress

**Fig. 2:**
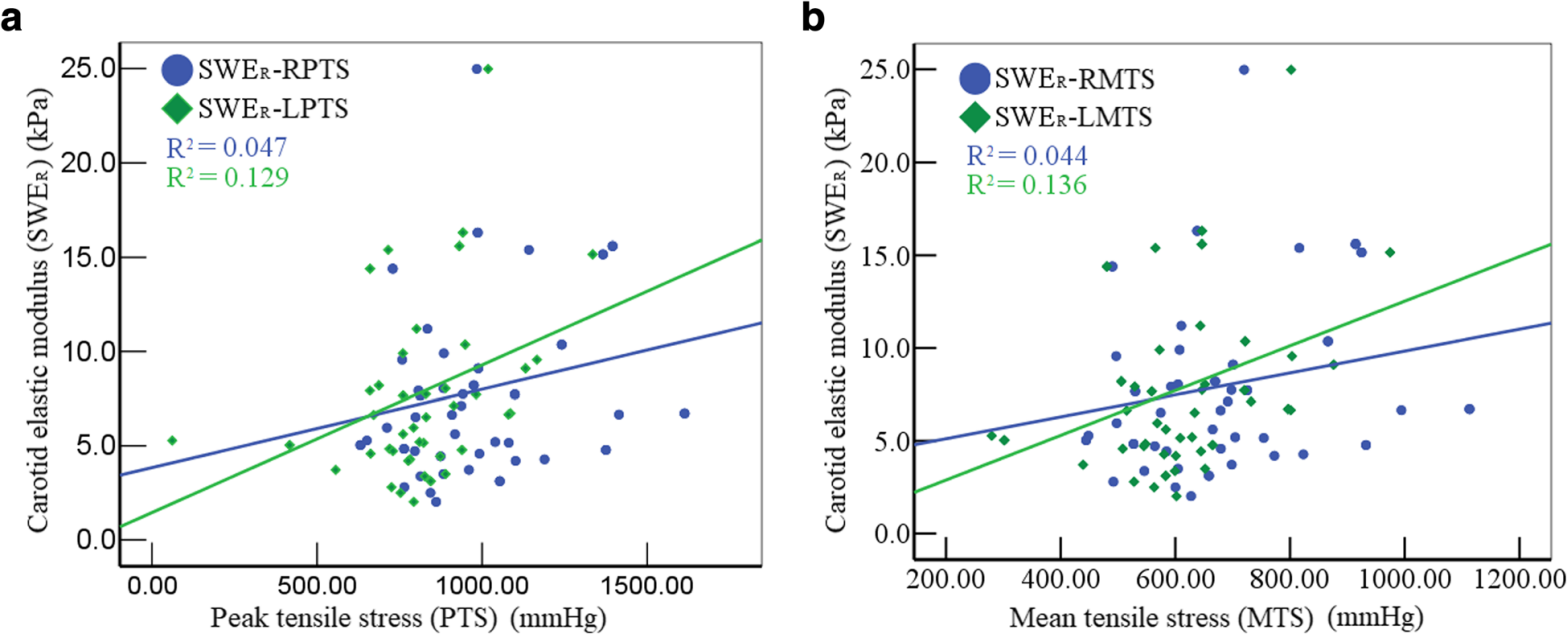
Correlation between the carotid viscoelasticity and tensile stress. **a** SWE_R_ correlated positively with right and left carotid peak tensile stress, respectively. **b** SWE_R_ correlated positively with right and left carotid mean tensile stress, respectively

Figure 2 and Table 3 showed that SWE_R_ has a positive correlation with R-PTS, R- MTS, L-PTS and L-MTS (*r* = 0.218, 0.359, 0.209,0.369, respectively, all *P*<0.05), whereas no correlation was found between the SWD_R_ and the parameters of TS (all *P*>0.05).

**Table 3:** Correlation analysis between carotid viscoelasticity tensile stress [*r*(*P*)]

Variable	SWE_R_			SWD_R_		
	All subjects	Group A	Group B	All subjects	Group A	Group B
R-PTS	0.218 (0.021)	0.137 (0.313)	0.159 (0.241)	0.096 (0.314)	−0.404 (0.002)	0.172 (0.204)
R-MTS	0.359 (0.001)	0.276 (0.040)	0.353 (0.008)	0.054 (0.568)	0.068 (0.619)	−0.062 (0.650)
L-PTS	0.209 (0.027)	0.196 (0148)	0.142 (0.298)	0.067 (0.485)	−0.352 (0.008)	0.153 (0.259)
L-MTS	0.369 (0.001)	0.415 (0.001)	0.313 (0.019)	0.005 (0.956)	0.086 (0.530)	−0.098 (0.474)

*SWE*_*R*_ Shear wave elastic modulus in electrocardiographic R wave, *SWD*_*R*_ Shear wave dispersion in electrocardiographic R wave, *R-PTS and R-MTS* Right carotid peak tensile stress and mean tensile stress, *L-PTS and L-MTS* Left carotid peak tensile stress and mean tensile stress

### Reproducibility analysis

**Fig. 3:**
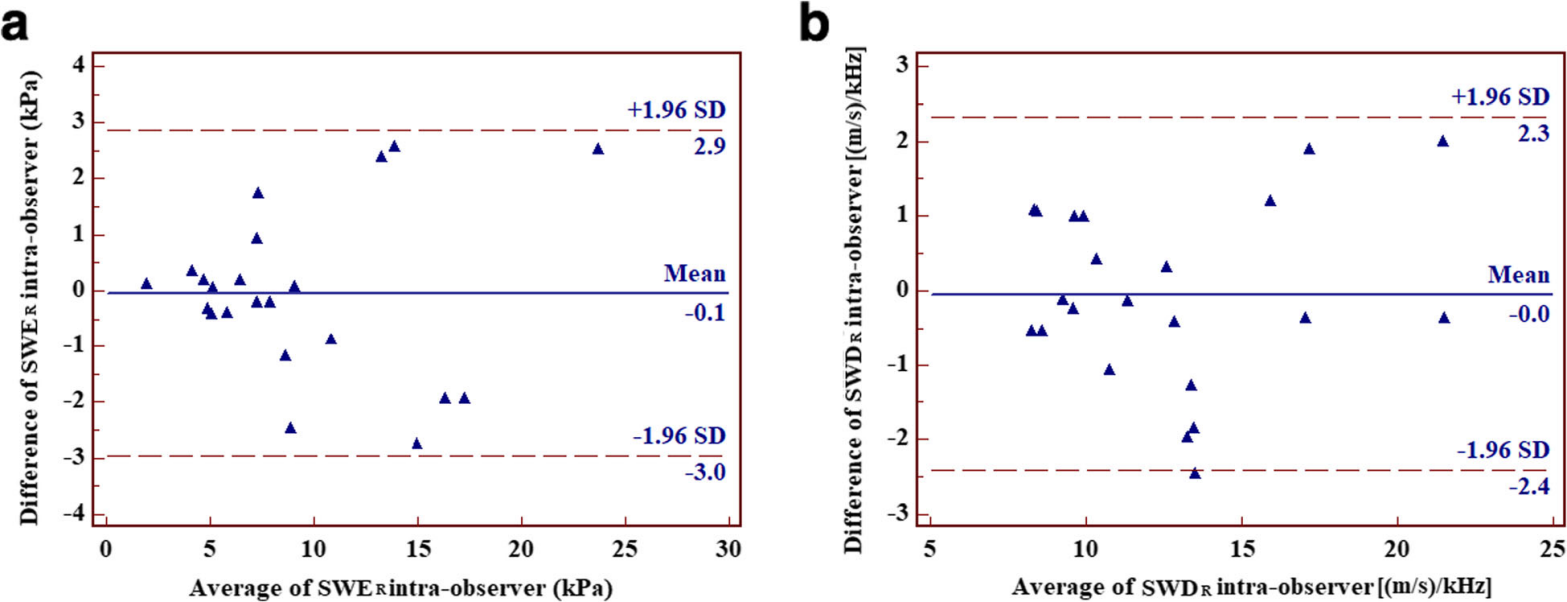
Bland-Altman plots for agreement in SWE_R_ (**a**) and SWD_R_ (**b**) measurement

A week later, a total of 22 subjects were randomly selected and involved in the reproducibility evaluation. SWD_R_ and SWE_R_ were measured repeatedly by the same investigator. The Bland–Altman plots showed the good agreement between the parameters obtained by two separate measurements (Fig. 3).

## Discussion

The cardiovascular and cerebrovascular diseases are still the number 1 cause of death globally. However, it is known that the onset and progression of these diseases can be predicted by the biomechanical features of large arteries, such as carotid arteries. Therefore, some of the predictors, such as mechanical stress and strain are very important for identifying the potential risk over the processes of arterial pathologies. TS, a parameter derived from certain measurements by using Laplace’s law, is associated with the inflation pressure, wall thickness and inner diameter [12]. In addition, TS of arterial wall is significantly related to the viscoelasticity of arteries. Our study shows that SWE_R_ and SWD_R_, the two parameters that represent the viscoelasticity of arteries were lower in the subjects older than 50 years that those of the younger subjects. We also found that no significant difference of PTS and MTS exist between the two groups, but the PTS of the right carotid arteries in the group with older ages was lower than the younger ones. The PTS and MTS, were positively corelated with SWE_R_, but no correlation was found between PTS and SWD_R_, which implys that the arterial elasticity may have some implication in the change of tensile stress.

The geometry changes of the carotid artery are closely related to the mechanical properties. CIMT, which is usually measured by noninvasive ultrasound, is strongly associated with cardiovascular and cerebrovascular events and used for evaluating progression: regression of atherosclerosis and for predicting arterial remolding and subsequent clinical complications [13, 14]. We measured the CIMT using radiofrequency-based ultrasound technique. The two-dimensional vascular structure can be clearly visualized, which allows the real-time measurement of CIMT within six cardiac cycles, with a resolution of 10 μm. This technology a reliable method for the clinical evaluation of arterial structure and function [15]. Our study shows that the CIMT increased in the subjects older than 50 years, suggesting that the carotid arterial remolding has some relationship with ages. CIMT measurements respectively made on the left and right carotid arteries could represent separate phenotypes because their patterns of associations with risk factors are different. For example, on the left carotid artery, the CIMT is thicker and shows stronger associations with blood lipid, glucose and lower estrogens; while on the right carotid artery, the CIMT is thinner and significantly related to hemodynamics, such as hypertension and heart rate. These results suggest that the weights of risk factors are different on left and right carotid arteries [16, 17]. In this study, the CCID was enlarged in the older ones, while CCAD, i.e. the difference of CCID between diastole and systole was decreased. These results also exhibited that carotid artery occurred remodeling with age.

The study by Carallo et al. [18] demonstrated that the circumferential wall tension (WT) of carotid arteries significantly increased with age. Conversely, the present results showed the TS did not parallel the increase in CIMT and CCID. The PTS of right carotid arteries is lower in the group with older ages, and no remarkable difference was found for the parameters of bilateral MTS and left PTS. Various reasons for participating in that: (1) Mechanical models of artery, such as WT and TS, derived from Laplace’s law can be used to relate the arterial inner radius (*r*) and internal pressure (*P*). The WT was defined as *P* × *r* [19]. The WT model assumes a very thin wall, and then handles the pressure, which does not take into account wall thickness. The TS, being a corrected Laplace model, was *P* × *r/CIMT*. TS could reflect the tensile response in circumference [11]. (2) The mechanical stretch can induce structural changes in the arterial wall, including VSMC hyperplasia and hypertrophy, as well as increased deposition of ECM collagen and elastin and result in arterial remodeling [20, 21]. On the other hand, the arterial remodeling could act on its mechanical properties [22, 23]. (3) The arterial tissue is viscoelasticity and show non-linear.

However, the arteries are of viscoelastic properties and exhibit the nonlinear stress-stain relations [5]. Several new non-invasive techniques have been used to study arterial elasticity, such as dimensional speckle-tracking imaging [24], ultrasonic radiofrequency tracking [25] and shear wave elastography [26–28]. However, in vivo, it is difficult to study the arterial viscidity due to its complex temporal changing behavior. Shear wave elasticity imaging may noninvasively evaluate the properties of soft tissues based on a group shear wave speed assuming that tissue is elastic; however, soft tissues are known to be viscoelastic, meaning the shear wave speed is dependent on the wave’s frequency content. Over the last years, there has been significant innovation in the area of describing the viscoelastic properties of soft tissue by the frequency-dependent: shear wave dispersion (SWD, the change in speed with frequency) [29]. In this work, the viscoelastic properties of carotid artery were evaluated by SWD. The SWE_R_ and SWD_R_ decreased with age. In addition, the TSs were positively connected with SWE_R_, while were not related to SWD_R_. This suggested that the arterial elasticity contributed its mechanical behavior rather than viscidity. The vascular smooth muscle cells, extracellular matrix proteins collagen and elastin play a crucial role in the viscoelastic properties, i.e. their spatial organization and interaction dominate the macroscopic non-linear vessel properties [5, 30]. Higher vascular stiffness is typically found in older subjects because the elastic lamellae decreases with age, while the connective tissue and collagen fibers increase [31]. The mechanical characteristics of arteries were related to local pathologies of the arterial system, while wall viscosity change reflects a more general influence of age and diseases [32].

There are limitations for this study. We included a small sample size of 45 subjects in this study. In addition, the curved abdominal transducer was used to evaluate the carotid viscoelasticity, while transducer of linear array could provide better images and measurements. We only include the subjects with healthy carotid arteries. In the future, we will explore the value of using this technique to characterize the tensile stress features of the carotid arteries with pathology, such as atherosclerosis.

## Conclusion

Ultrasonic shear wave imaging could be used to quantitatively assess carotid viscoelasticity. The tensile stress of carotid arteries is closely related to the elastic properties of aging carotid arteries, but no apparent correlation with the viscosity of carotid arteries, suggesting that mechanical properties of the arterial wall with age might be better revealed.

## Data Availability

The datasets are analyzed and are not publicly available, but are available from the corresponding author on reasonable request.

## Abbreviations

CCA: Common carotid artery
CCID: Common carotid inner diameter
CIMT: Common carotid intima-media thickness
DBP: Diastolic blood pressure
LDL: Low-density lipoprotein-cholesterol
MTS: Mean tensile stress
PTS: Peak tensile stress
SBP: Systolic blood pressure
SWD: Shear wave dispersion
SWD_R_: Shear wave dispersion in electrocardiographic R wave
SWE: Shear wave elastography
SWE_R_: Shear wave elastic modulus in electrocardiographic R wave
TC: Total cholesterol
TG: Triglycerides
TS: Tensile stress
WT: Wall tension
